# Development and Evaluation of an Eco-Friendly Hand Sanitizer Formulation Valorized from Fruit Peels

**DOI:** 10.1155/2023/2516233

**Published:** 2023-12-30

**Authors:** J. Verma, R. Mishra, A. Mazumdar, R. Singh, N. Sh. El-Gendy

**Affiliations:** ^1^Department of Biotechnology, Noida Institute of Engineering and Technology (NIET), Plot No. 19, Knowledge Park II, Institutional Area, Greater Noida, UP-201306, India; ^2^Noida Institute of Engineering and Technology, Pharmacy Institute, Plot No. 19, Knowledge Park II, Institutional Area, Greater Noida, UP-201306, India; ^3^Lovely Professional University, Phagwara, Punjab 144001, India; ^4^Egyptian Petroleum Research Institute (EPRI), Nasr City, Cairo, P.O. 11727, Egypt; ^5^Center of Excellence, October University for Modern Sciences and Arts (MSA), 6th of October City, Giza, P.O. 12566, Egypt

## Abstract

Hand sanitizer usage has proven to be a common and practical method for reducing the spread of infectious diseases which can be caused by many harmful pathogens. There is a need for alcohol-free hand sanitizers because most hand sanitizers on the market are alcohol-based, and regular use of them can damage the skin and can be hazardous. India is the world's largest producer of fruits and one of the major problems after fruit consumption is their peels, causing waste management problems and contributing to the formation of greenhouse gases leading to air pollution and adding to the problem of climate change. Valorization of such wastes into other value-added products and their incorporation into formulations of eco-friendly alcohol-free hand sanitizers would solve these issues, save the environment, benefit the society, and help in achieving the sustainable development goals. Thus, this research focuses on formulating an effective natural alcohol-free hand sanitizer that harnesses the antimicrobial properties of the various types of bioactive components found in fruit peels of pomegranate, sweet lime, and lemon. The peel extracts and the formulated sanitizer proved considerable antimicrobial activity against the pathogenic *Escherichia coli* and hand microflora. Molecular docking was also applied to examine ligand-protein interaction patterns and predict binding conformers and affinity of the sanitizer phytocompounds towards target proteins in COVID-19, influenza, and pneumonia viruses. The binding affinities and the protein-ligand interactions virtual studies revealed that the sanitizer phytocompounds bind with the amino acids in the target proteins' active sites via hydrogen bonding interactions. As a result, it is possible to formulate a natural, alcohol-free hand sanitizer from fruit peels that is effective against pathogenic germs and viruses using the basic structure of these potential findings.

## 1. Introduction

Hand sanitizer usage is the simplest and the most convenient method to stop the transmission of microbes and infections, particularly those that are “enteric” or airborne. These infections can be transmitted by contact with contaminated surfaces as well as by droplets released from the mouth and nose when coughing, sneezing, or talking by an infected person. When we use hand sanitizer, it kills or inactivates any microorganisms that might be on our hands [1].

Hand sanitizers are essential for keeping our hands germ-free, but the chemicals in many commercial products can be harsh on our skin and the environment [2]. The use of herbal hand sanitizers has become increasingly popular in recent years. Herbal products offer natural and organic alternatives to traditional, chemical-based hand sanitizers. Hand sanitizers come in a variety of compositions, including liquid, foam, and simple gel [3]. Hand sanitizers are quick, simple, portable, and handy to use. According to several studies, families who use hand sanitizers have a lower chance of transmitting respiratory and gastrointestinal infections [4]. Incorrect use of the chemical-based sanitizers may result in toxicity in humans and the environment. A higher risk of various viral illnesses and antibiotic resistance has also been associated with frequent usage of hand sanitizers [5]. Traditional alcohol-based sanitizers, while effective at killing germs and preventing infection transmission, might have environmental consequences that must be considered. They have a substantial impact on the environment, and their manufacturing and use have been connected to a variety of health issues, including skin irritation, dryness, and accidental ingestion [6]. Plant-based sanitizers, especially those derived from fruit peels, provide an alternative solution to these environmental challenges. The following show how they can help [7]:

- Biodegradability: plant-based sanitizers usually contain natural ingredients derived from sustainable plant sources. These materials are less harmful to the environment and more biodegradable than the chemical-based ones found in the conventional sanitizers. Thus, this would protect life on land and help in achieving the SDG15.
- Natural and renewable resources: the use of fruit peel pats into natural and renewable resources, promoting sustainability. By utilizing by-products that might otherwise go to waste, the formulation contributes to the circular economy and minimizes environmental impact. Thus, this would help in achieving SDG11 of sustainable communities and cities.
- Reduced environmental footprint: compared to the production of synthetic ingredients or the chemical extraction of certain plant extracts, utilizing nonalcoholic fruit peel extract may have a lower environmental footprint, achieving SDG12, i.e., responsible for consumption and production. This can be attributed to the reduced energy consumption and fewer chemical processes involved.
- Solving the waste management problem: the valorization of fruit peels into hand sanitizer contributes to the reduction of persistent environmental pollutants, achieving the SDG9 related to industry, innovation, and infrastructure.
- Lower carbon footprint: fruit peel-based sanitizer has the potential to minimize the greenhouse gas (GHG) emissions and the overall carbon footprint. Instead of leaving fruit peels to be degraded emitting methane and other GHG emissions, it is valorized into a value-added product, so it would help in the mitigation of climate change problems, thus achieving the SDG13.
- Skin-friendly formulations: fruit peel-based sanitizers frequently include natural moisturising agents and skin-friendly components, which reduce the risk of skin irritation and dryness associated with frequent sanitizer usage, so it would help in achieving the SDG3 of good health and well-being.
- Alignment with corporate social responsibility (CSR): emphasizing the sustainability aspect of the product aligns with corporate social responsibility goals. It positions the brand as environmentally conscious and socially responsible, which can be attractive to consumers. Thus, it would enhance the actions towards the green economy.

The term “phytochemicals” derived from the Greek word “phyto” which means “plant” describes bioactive, nonnutritive chemical substances that are present in plants, which have a variety of health-promoting effects [8]. These qualities include the ability to lower cholesterol, the ability to reduce platelet aggregation, the ability to manage hormone metabolic processes, antitumor properties, antioxidant activity, antimicrobial action, modulation of detoxification enzymes, and activation of the immune system [9]. Secondary metabolites found in abundance in plants, including tannins, terpenoids, and alkaloids, have been shown to exhibit *in vitro* antibacterial activities [10]. There is evidence that plant and fruit extracts contain antibacterial, antifungal, and antiviral properties [11]. Antimicrobial activity reports are in studies of pomegranate, orange, and lemon using the disc diffusion method, but there is little information on the growth kinetics of pathogens with respect to these different extracts of the fruits [12]. Many secondary metabolites found in fruit peels have been reported to have antimicrobial, antiviral, and antifungal properties, including phenolic compounds, tannins, terpenoids, alkaloids, and flavonoids [13, 14]. The synthesis and the study of the biologically active substances of the plant begin with extraction, which is the most crucial step. The most effective extraction technique is analytical, quick, and nondestructive. Due to the ease of access to water and lesser toxicity, the traditional method for using medicinal plants was to ingest the extracts in food or by boiling them in water [15]. The conventional solvent extraction (CSE) method is used to recover bioactive compounds [16]. However, it has a number of disadvantages, including excessive solvent consumption, hazardous liquid organic solvents, lengthy extraction durations, and volatilizing. The extracts obtained through CSE were comparable in terms of levels of polyphenols to those extracted using unconventional techniques as demonstrated in the study [17]. The total phenolic content is reported to be significantly influenced by the properties of extraction solvents [1]. The phenolic compounds are easily dissolved by high polarity solvents, such as methanol; however, it is one of the hazardous solvents, but it can also be performed by ethanol, which is considered a food-grade solvent [1].

**Figure 1:**
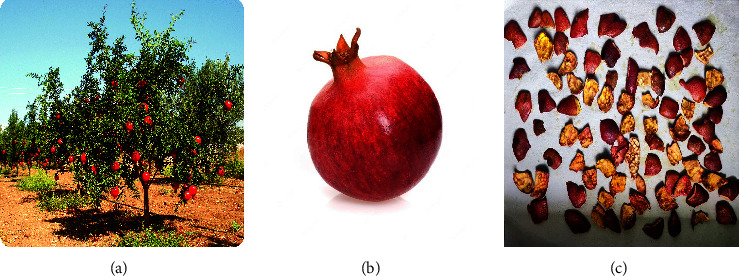
*P. granatum* fully grown tree (a), fruit (b), and fruit peels (c).

The pomegranate (*Punica granatum*) (Figure 1) is extensively cultivated across Asia, North Africa, the Mediterranean region, and the Middle East. Pomegranate peels have been used for centuries in conventional medicine in America, Asia, Africa, and Europe to treat various ailments [18]. Pomegranate peel is a by-product of the fruit juice manufacturing business, accounting for around 30%–40% of the fruit component [19]. Pomegranate phytochemistry has been extensively researched, and pomegranates are discovered to be a rich source of polyphenolic compounds (Table 1) [23]. The *P. granatum* peels contain significant amounts of flavonoids and tannins (Table 1). Pomegranates are rich in antioxidants, which help protect cells from damage. Their antimicrobial activity has been studied extensively, and they demonstrated effectiveness against a number of bacteria and fungi. Secondary metabolites in pomegranate peel include flavonoids, ellagic acid, proanthocyanins, minerals, tannins, anthocyanins, and polyphenolic compounds (Table 1) [20]. Punicalagin is the primary active compound responsible for the antimicrobial activity of pomegranate peels. Moreover, pomegranate exhibits antimicrobial, antifungal, antiviral, vermifugal, antiparasitic, and molluscicidal properties [24].

**Figure 2:**
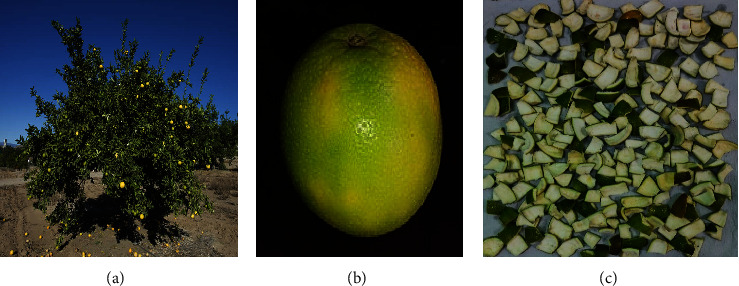
*C. limetta* fully grown tree (a), fruit (b), and fruit peels (c).

The sweet lime (Figure 2), commonly referred to as “mosambi” originates from Asia and is most commonly grown in India, China, southern Japan, Vietnam, Malaysia, Indonesia, and Thailand. The peel of sweet lime represents 57% of the fruit and its extract exhibits antimicrobial properties against various pathogens. Limonene, geraniol, and linalool [21] are the major active compounds responsible for these activities. The most abundant terpene, d-limonene (Table 1), has antimicrobial properties, most notably the demonstration of antibacterial properties against Gram-positive bacteria. It also enhances the efficacy of sodium benzoate as a preservative [25]. Sweet lime is a valuable crop for farmers because it requires less water and is more tolerant of heat and drought than other citrus fruits. Furthermore, the fruit is high in nutrients and has numerous health benefits, making it a popular choice among consumers. Sweet lime peel has been investigated for its ability to act as a natural antibacterial agent in a variety of applications, including hand sanitizers [26]. Furthermore, the sweet lime industry produces by-products including sweet lime peel, which can be utilized to extract bioactive chemicals with potential applications in the culinary, pharmaceutical, and cosmetic industries [16].

**Figure 3:**
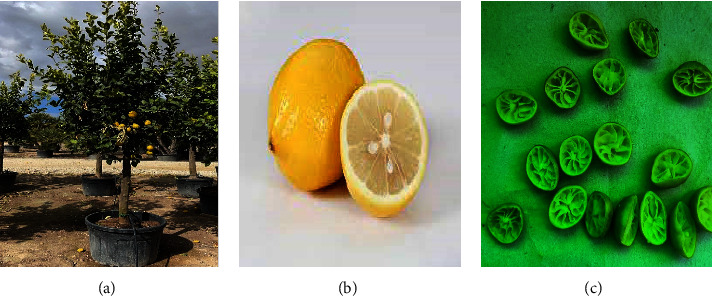
*C. limon* fully grown tree (a), fruit (b), and fruit peels (c).

Lemon (*C. limon* (L.) Osbeck, Figure 3) is a *Rutaceae* family medicinal plant. It is mostly cultivated for its alkaloids, which have antimicrobial properties against clinically significant strains of bacteria in crude extracts of different sections of the lemon (more particularly, leaves, stems, roots, and flowers) [27, 28]. Lemon peels, which represent approximately 50% of lemon fruit, are known to exhibit antimicrobial activity against many pathogens and are also rich in antioxidants, vitamins, and flavonoids [22]. The primary bioactive components found in lemon peels are limonene, citral, and linalool (Table 1) [29].

The coronavirus disease, also known as COVID-19, is a highly contagious respiratory ailment caused by the new coronavirus SARS-CoV-2 [30]. It was discovered in December 2019 in Wuhan, China, and quickly spread throughout the world, resulting in a global pandemic [30]. When an infected person coughs, sneezes, talks, or breathes, the virus spreads mostly through respiratory droplets [31]. Contacting contaminated surfaces and then contacting the face might also spread it [31]. Various public health interventions have been implemented worldwide to restrict the spread of COVID-19 [30, 31]. Widespread testing, contact tracking, quarantine and isolation methods, social distancing, mask use, and promotion of excellent hand hygiene are among them. Vaccines have also been produced and distributed widely, protecting against severe sickness and lowering the virus spread [30, 31].

The flu, or influenza, is an infectious respiratory illness [31]. It is caused by influenza viruses, the most prevalent of which are types A, B, C, and D [31]. Influenza symptoms range from mild to severe and often include fever, cough, sore throat, runny or stuffy nose, muscular or body aches, headaches, and exhaustion. It can spread in the same ways as COVID-19 [30]. Preventive practices including frequent hand-washing, using tissues or elbows to cover coughs and sneezes, and avoiding close contact with sick people are critical in limiting viral spread [32].

Pneumonia is a lung infection that causes inflammation in the air sacs known as alveoli [33]. Pneumonia can be caused by a variety of pathogens, including bacteria, fungi, and parasites [34]. Respiratory viruses responsible for the viral pneumonia are influenza viruses, including, H1N1 and H3N2, respiratory syncytial virus (RSV), adenovirus, rhinovirus, and coronaviruses, including, SARS-CoV-2, the virus responsible for COVID-19 [34]. These viruses can infiltrate the respiratory tract and cause a lung infection [35]. When an infected individual coughs or sneezes, viral pneumonia spreads through respiratory droplets [30]. Practicing basic respiratory hygiene, such as covering the mouth and nose when coughing or sneezing, washing hands often, and avoiding close contact with others who have respiratory infections, can help prevent viral pneumonia [31].

Although herbal hand sanitizers are known to be highly safe, effective, convenient, eco-friendly, affordable, and most effective in killing germs, they have some limitations which are as follows [35]:

- Effectiveness against certain pathogens: it may not be effective at eliminating certain pathogens, such as norovirus, compared to the conventional alcohol-based sanitizers
- Some herbal hand sanitizers may not be effective for a long time and may need to be used more frequently
- The quality and effectiveness of herbal hand sanitizers may vary depending on the source and quality of the plant extracts utilized
- Consistency and stability: herbal formulations may lack consistency and stability over time, leading to variations in their effectiveness. The shelf life of herbal hand sanitizers may be shorter compared to synthetic formulations. It is essential to consider the stability of the product to ensure its efficacy

**Scheme 1:**
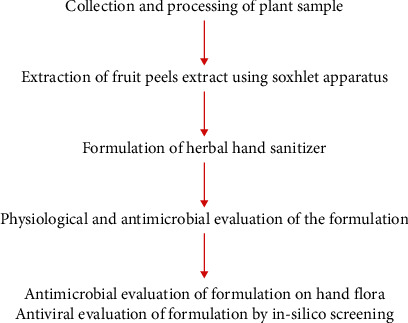
Workflow of the current research.

The ultimate goal of this research is to contribute to the development of a natural and safe alternative to conventional alcohol-based hand sanitizers, promoting sustainable and eco-friendly hygiene practices. The study focuses on formulating an eco-friendly and effective alcohol-free hand sanitizer that harnesses the antimicrobial properties of the various types of bioactive components found in fruit peels. Pomegranate (*Punica granatum*) peels, lemon (*Citrus limon*) peels, and sweet lime (*Citrus limetta*) peels, containing active phytocompounds with antimicrobial properties, are valorized in this current study to prepare an alcohol-free sanitizer. The research involves extraction methods, formulation development, evaluation of the sanitizer's physical and antimicrobial properties, and assessment of its efficacy in reducing microbial load on the hands (Scheme 1). The phytocompounds present in fruit peel extracts formulating the sanitizer are also virtually studied for antiviral activity via molecular docking against COVID-19, influenza, and pneumonia viruses.

## 2. Materials and Methods

### 2.1. Collection and Processing of Sample

Peels of pomegranate, sweet lime, and lemon were collected from the local market from various fruit juice vendors. The peels were cleaned and washed thoroughly under tap water and then dried in shade at room temperature for 2-3 days. After that, the peels were shredded into smaller parts and left for drying. The dried peels were weighed and powdered by using a mortar and pestle.

### 2.2. Phytochemicals Extraction and Sanitizer Preparation

**Figure 4:**
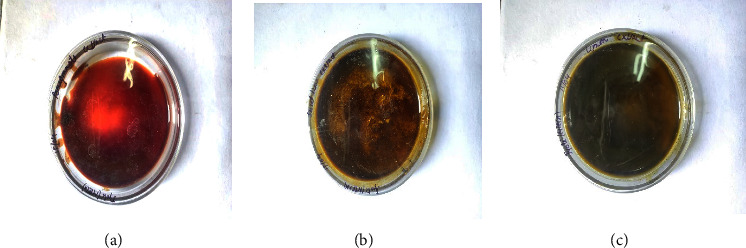
Pomegranate extract (a), sweet lime peel extract (b), and lemon peel extract (c).

**Figure 5:**
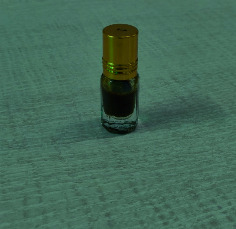
Formulated herbal hand sanitizer in a screw-capped glass bottle.

20 g of powdered peels were transferred in a 250 mL round flask of a Soxhlet apparatus containing 150 mL of ethanol and then heated for 6 hrs at 60°C. A rotary evaporator set at 78°C and 20 kPa was used to obtain a concentrated extract and to recycle ethanol for reusability. The extract yield (Figure 4) was calculated. The concentrated extracts were then equally mixed and glycerine was added in a final formulation as listed in Table 2. The obtained mixture was then vigorously mixed to get a homogenous mixture. Finally, the prepared sanitizer was stored in a screw-capped glass bottle (Figure 5) at room temperature for further analysis.

### 2.3. Physical and Antimicrobial Evaluation

The pH was measured by a pH meter (Digimed model DM-22, São Paulo, Brazil). Brookfield DV1M Viscometer (Labomat Essor, Saint-Denis, France) and pycnometer were used to measure viscosity and density at room temperature. The color and odor of the prepared sanitizer were also evaluated. The antimicrobial evaluation was performed against the pathogenic *Escherichia coli* ATCC 23282 and real sampled hand microflora.

#### 2.3.1. Disc Diffusion Method

Discs of 6 mm diameter were made from Whatman filter paper. Discs were autoclaved at 120°C and 1.2 bar for 15 min. The sterilized discs were then dipped separately for 2 min into glycerine (–ve control) and the prepared sanitizers (peel extracts). 100 *μ*L of *Escherichia coli* ATCC 23282 were inoculated onto sterile nutrient agar (SNA) plates by a spreader. Then, the prepared discs were placed onto the SNA plates under aseptic conditions and incubated at 37°C for 24 hrs. At the end of the incubation period, the plates were checked to measure the zone of inhibition (ZoI) which was measured in millimetres.

#### 2.3.2. Antimicrobial Evaluation of Hand Microflora

Sterilized cotton swab at 120°C and 1.2 bar for 15 min was rubbed thoroughly against hand samples (from both the left and right hands). Then, that contaminated swab was put in test tubes of nutrient broth. The test tube of nutrient broth was incubated for 24 hrs at 37°C. At the end of the incubation period, serial dilutions (10^−1^) of each broth culture were spread onto SNA plates to obtain pure isolates. Obtained pure isolates were maintained and cultured in broth media. The disc diffusion method was applied to test the antimicrobial effect of the prepared sanitizer on isolated microorganisms. Glycerine was used as the negative control.

All experiments were performed in triplicates and the average results with a standard deviation (StD) range of ±0.5% were tabulated. *p* < 0.05 was considered statistically significant at *α* = 0.05 level with a 95% confidence interval.

### 2.4. In Silico Study of the Antiviral Activity of the Prepared Hand Sanitizer

Molecular docking, which is a bioinformatics-based theoretical simulation technique was applied for the in silico study to examine the ligand-protein interaction patterns and predict the binding conformers and affinity.

#### 2.4.1. AutoDock Vina

AutoDock Vina 1.1.2 is a popular molecular docking software tool for predicting the binding mechanism and affinity of small ligands (such as drug molecules) to protein targets. Oleg Trott founded AutoDock Vina 1.1.2 in the Molecular Graphics Laboratory at the Scripps Research Institute in 2010 [36]. It is a new and free tool for molecular docking, drug development, and virtual screening. It also provides high performance, multicore competency, increased precision, and a straightforward handling technique. AutoDock Vina 1.1.2 generates the grid maps and clusters. AutoDock Vina 1.1.2 has been shown to predict more accurate results than other methods [37].

#### 2.4.2. Steps in the Process of AutoDock Vina

1. Choosing the target protein and the ligand
2. Defining binding site and grid box for docking
3. Preparing the configuration file
4. Performing docking operations and visualizing the output

In this work, 14 phytochemical compounds (Table 3) that are known to be found in pomegranate, sweet lime, and lemon peel extracts were chosen to study their potential for the prevention of COVID-19, influenza virus, and *Streptococcus pneumoniae*. These compounds were chosen based on their potential medicinal properties and previous research indicating their effectiveness against these diseases [30, 32, 33, 37]. In addition, standard compounds were included, namely, remdesivir native (N3) for COVID-19, peramivir for influenza virus, and penicillin-G, L-amoxicillin, and cefprozil for *Streptococcus pneumoniae*. These standard compounds serve as reference points for comparison and validation of the virtual screening results.

**Figure 6:**
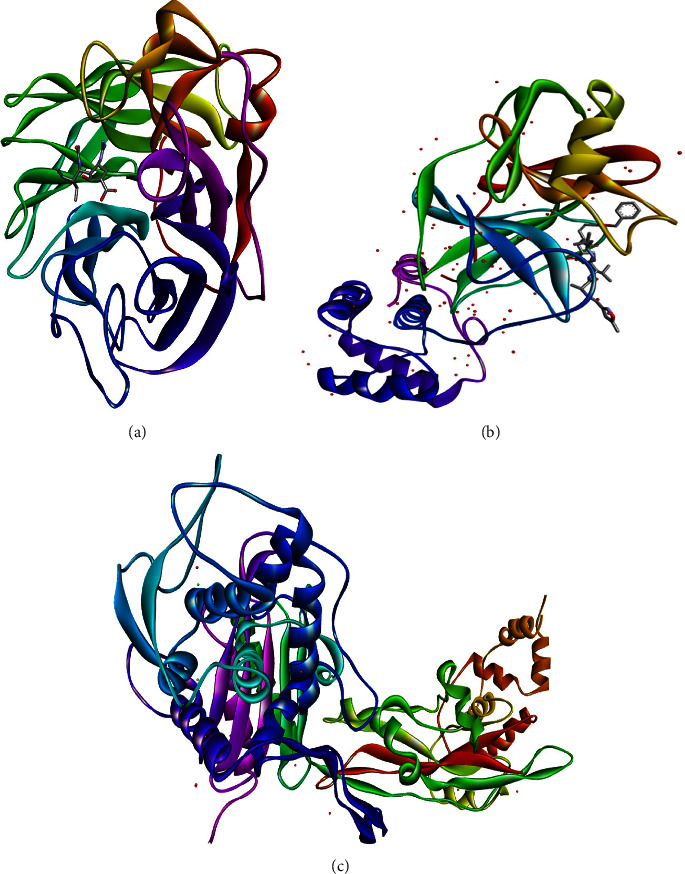
3D structure of N8 neuraminidase (PDB: 2HTU) (a), COVID-19 main protease (PDB: 6LU7) (b), and penicillin-binding protein 2B (PBP2B) from *Streptococcus pneumoniae* (strain R6) (PDB: 2WAF).

The major N8 neuraminidase (2HTU) for influenza (Figure 6(a)), protease (6LU7) for COVID-19 (Figure 6(b)), and penicillin-binding protein 2B (PBP2B) from *Streptococcus pneumoniae* (strain R6) (PDB: 2WAF) (Figure 6(c)) were chosen for this study. Then, ligands specific to this target were selected from the IMPPAT database (https://cb.imsc.res.in/imppat/) and the files were accordingly processed for virtual screening. Molecular docking using AutoDock Vina 1.1.2 was used to evaluate the binding affinities of the ligands against each target.

The binding energy of all the 14 compounds as well as the standard treatments was calculated and presented. Molecular interaction studies were also conducted to analyse the binding affinities and hydrogen bond interactions of various compounds against COVID-19, *Streptococcus pneumoniae'*s penicillin-binding protein 2B (PBP2B), and neuraminidase protein of the influenza virus.

## 3. Results and Discussion

### 3.1. Extract Yields and Physical Characteristics of the Formulated Sanitizer

The pomegranate (*Punica granatum*) peels, sweet lime (*Citrus limetta*) peels, and lemon (*Citrus limon*) peels yielded 6.4, 10.8, and 9.5 g of phytochemical extract/20 g peels, respectively (Table 4). There is a high statistical difference between the yield obtained from pomegranate peels relative to both obtained from lemon and sweet lime peels (*p* < 0.0001). However, there is no high statistically significant difference in extract yields obtained from lemon and sweet lime peels (*p*=0.03).

It has a forest green color (Figure 5), with a pH of 6, and a citrus woody odor. Thus, the formulated sanitizer can be safely applied on the skin, as it has been previously reported sanitizers with pH ranges of 4–7 are considered to be safe on human skin [6]. The formulated sanitizer recorded 0.953 g/mL density and 1.12 cP viscosity. Thus, the sanitizer can be easily tipped and seeped on hand.

### 3.2. Antimicrobial Evaluation

#### 3.2.1. Antimicrobial Evaluation of Different Fruit Peel Extracts against *E. coli*

**Figure 7:**
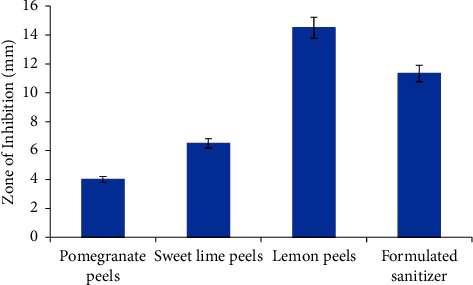
Antimicrobial activity of different peel extracts and formulated sanitizer against *E. coli*.

**Figure 8:**
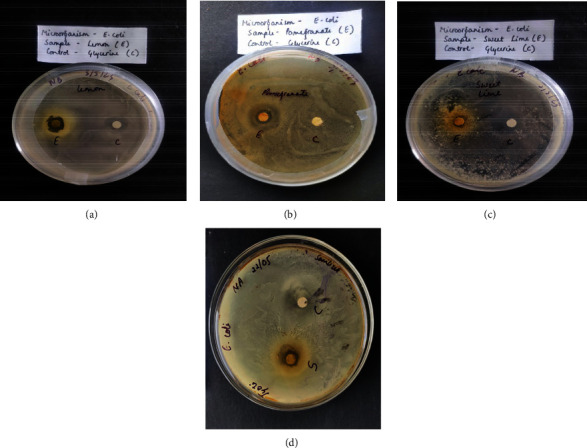
Antimicrobial activities of lemon peel extract (a), pomegranate peel extract (b), sweet lime peel extract (c), and formulated sanitizer (d) against *E. coli* in comparison with glycerine (−ve control).

The Gram-negative *E. coli* is known to be one of the physiological intestinal microflora and is multiresistant to several antibiotics leading to sepsis and wound infections when it comes outside the intestine [38]. The three peel extracts expressed sufficient antimicrobial effect against *E. coli*. The largest ZoI was recorded in NA plates injected with lemon peel extract (Figures 7 and 8), recording 14.5 ± 0.5 mm, with a high statistically significant difference compared with those recoded NA plates injected with other extracts (*p* < 0.0001). This was comparable with results reported by Otang and Afolayan [38]. There was a statistically significant difference (*p*=0.001) between the observed ZoI in NA plates injected with sweet lime peels and pomegranate peel extracts, recoding 6.5 ± 0.5 mm and 4 ± 0.5 mm, respectively (Figures 7 and 8). The phenols, alkaloids, tannins, terpenoids, and flavonoids in plant extracts are reported to have antimicrobial activities [13]. The presence of punicalagin and gallagic acid in the pomegranate peel extract might be the main reason for its antimicrobial effect [39]. The presence of d-limonene would be the main reason for the expressed antimicrobial activity of sweet lime and lemon peel extracts [40].

#### 3.2.2. Antimicrobial Evaluation of the Formulated Hand Sanitizer against *E. coli*

The formulated hand sanitizer expressed a relatively high antimicrobial efficiency against the Gram −ve *E. coli* (Figures 7 and 8), recording a ZoI of 11.33 ± 0.5 mm, with a high statistically significant difference (*p* < 0.0001) relative to those recorded in NA plates injected with different peel extracts. The recorded antimicrobial activity might be related to the phytochemical constituents of the peel extracts formulating the prepared hand sanitizer [20, 21, 23, 28]. Tannins can affect both microbial cell walls and membranes as they can precipitate proteins and negatively impact glycosyltransferases [38]. Polyphenols have the potential to impact bacterial cell walls, interfere with protein interactions, block enzymes through oxidised agents, and disrupt the coaggregation of microorganisms [38].

#### 3.2.3. Antimicrobial Evaluation of the Formulated Sanitizer against Hand Microflora

**Figure 9:**
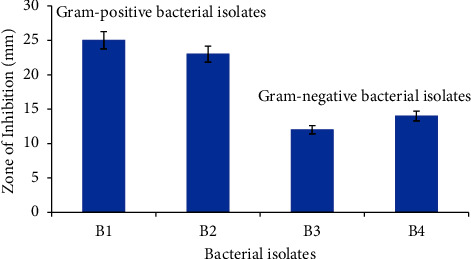
Antimicrobial activity of the formulated sanitizer against isolated hand microflora.

**Figure 10:**
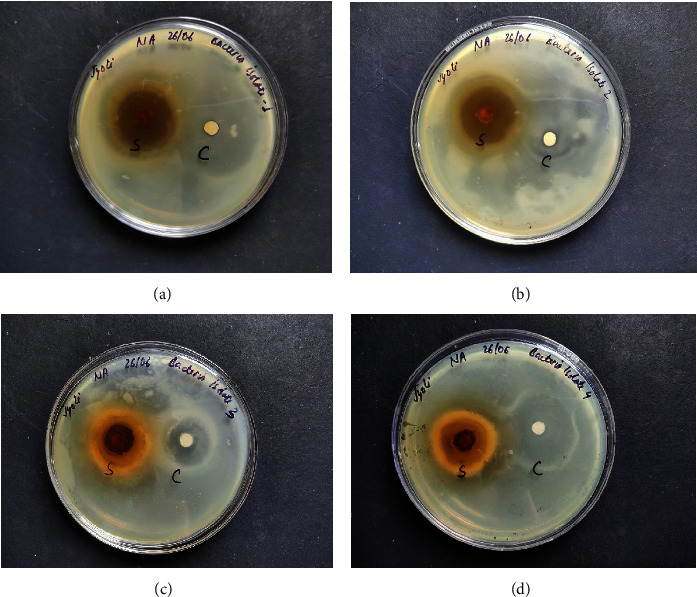
Antimicrobial testing of the formulated sanitizer (S) against the isolated hand microflora: the Gram-positive bacterial isolates (a, b) and the Gram-negative bacterial isolates (c, d) in comparison with glycerine (−ve control).

Gram-positive staphylococci (B1), rod-shaped bacilli (B2), and Gram-negative small rod-shaped motile bacilli (B3 and B4) were isolated from hand swap samples. The formulated sanitizer expressed efficient antimicrobial capabilities against the isolated Gram +ve and Gram −ve bacteria (Figures 9 and 10), with ZoI ranging between 12 ± 0.5 mm and 25 ± 0.5 mm. There was a high statistically significant difference between the antimicrobial activities of the formulated sanitizer against Gram −ve and Gram +ve bacterial isolates (*p* < 0.0001). The Gram −ve bacteria are known to be more resistant to antibiotics than Gram +ve ones, due to the presence of the protecting outer membrane layer with its protein, phospholipids, and lipopolysaccharide constituents [41].

### 3.3. Antiviral Evaluation by In Silico Screening

#### 3.3.1. Binding Affinity and the Molecular Docking Interactions of the Phytocompounds against Target Receptor of Viruses

The binding affinity is the strength of the interaction between two (or more than two) molecules that bind (i.e., interact) reversibly. The binding affinity is the key to appreciation of the intermolecular interactions driving biological processes, structural biology, and structure-function relationships [37]. The binding energy provides insights into the strength of the interaction between a compound and its target protein. Lower binding energy values (i.e., more negative values) indicate stronger interactions, suggesting the potential efficacy of the compound in inhibiting the target protein's function [37].

**Figure 11:**
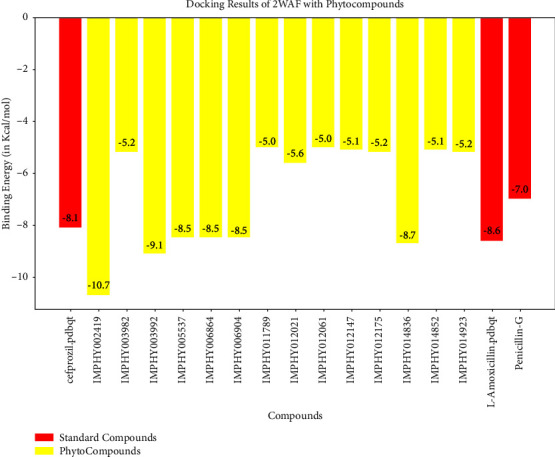
Molecular docking results showing binding energy of phytocompounds with standard compounds against pneumonia virus.

**Figure 12:**
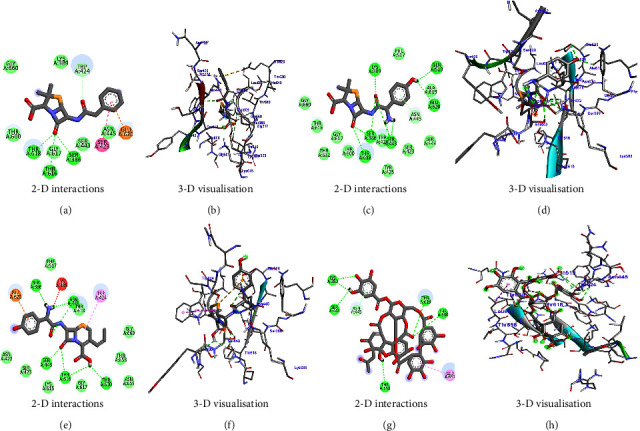
Suggested interaction between 2WAF and penicillin-G (a, b), 2WAF and L-amoxicillin (c, d), 2WAF and cefprozil (e, f), and 2WAF and IMPHY002419 (g, h).

Molecular docking using AutoDock Vina helps in evaluating the binding affinities of the ligands against each target. For *Streptococcus pneumoniae*-binding protein 2B (PBP2B), the standard compounds cefprozil, penicillin-G, and L-amoxicillin displayed binding energies of −8.1, −7.0, and −8.6 kcal/mol, respectively (Table 5 and Figure 11). Note that, the phytocompound IMPHY002419 (i.e., ellagitannins, Table 3) was identified with a superior binding energy of −10.7 kcal/mol (Table 5 and Figure 11), thus outperforming the three standard compounds.  Figure 12 shows the suggested interaction. Cefprozil is suggested to form hydrogen bond interactions with five amino acids: ASN A: 445, SER A: 386, SER A: 443, THR A: 616, and THR A: 600. L-Amoxicillin is suggested to form hydrogen bond interactions with six amino acids: THR A: 618, SER A: 386,SER A: 443, LYS A: 389, GLU A: 620, and GLN A: 519. Penicillin-G was also suggested to form hydrogen bond interactions with three amino acids: THR A: 618, THR A: 616, and SER A: 386. However, among the screened 14 phytocompounds, IMPHY002419, which displayed the notable high binding affinity, is suggested to form hydrogen bond interactions with five amino acids: THR A: 618, THR A: 630, GLN A: 519, ASN A: 445, and TRP A: 424. These findings suggest that the phytocompound IMPHY002419 exhibits a stronger binding affinity against PBP2B of *Streptococcus pneumoniae* compared to the standard compounds.

**Figure 13:**
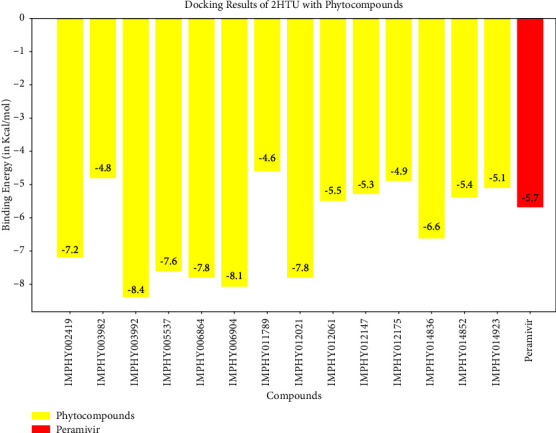
Molecular docking results showing binding energy of phytocompounds with standard compound against influenza virus.

**Figure 14:**
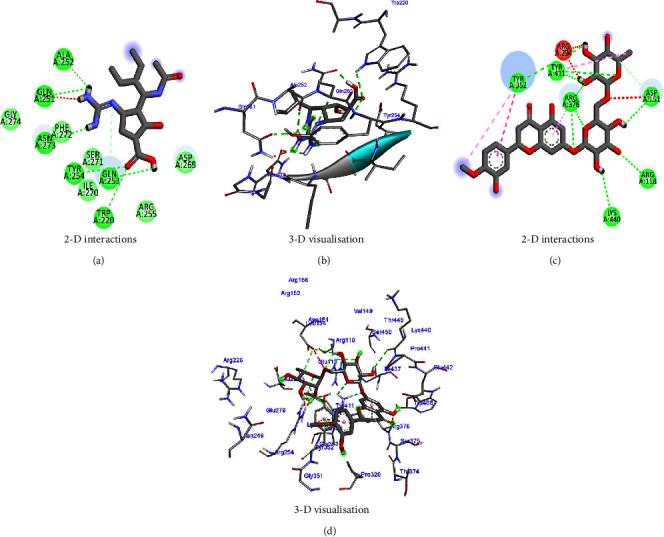
Suggested interaction between 2HTU and peramivir (a, b) and 2HTU and IMPHY003992 (c, d).

In the case of influenza, the standard compound peramivir showed a binding energy of −5.7 kcal/mol (Table 6 and Figure 13). However, the phytocompound IMPHY003992 (i.e., hesperidin Table 3) was found to express a higher binding energy of −8.4 kcal/mol (Table 6 and Figure 13), indicating a higher interaction than the standard compound.  Figure 14 shows the suggested interaction. The standard compound peramivir is suggested to form hydrogen bond interactions with six amino acids: ALA A: 252, GLN A: 251, ASN A: 273, TYR A: 254, TRP A: 220, and GLN A: 253. The phytocompound IMPHY003992 is suggested to form hydrogen bond interactions with six amino acids: TYR A: 411, TYR A: 352, ARG A: 376, ASP A: 151, ARG A: 118, and LYS A: 440. These findings suggest that the hydrogen bond interactions observed between the phytocompound IMPHY003992 and key amino acids contribute to the stability of the compound-protein complex, potentially enhancing its effectiveness as an inhibitor of neuraminidase [42]. All these molecular interactions play a crucial role in stabilizing the binding between the compounds and the protein, potentially influencing their effectiveness as therapeutic agents.

**Figure 15:**
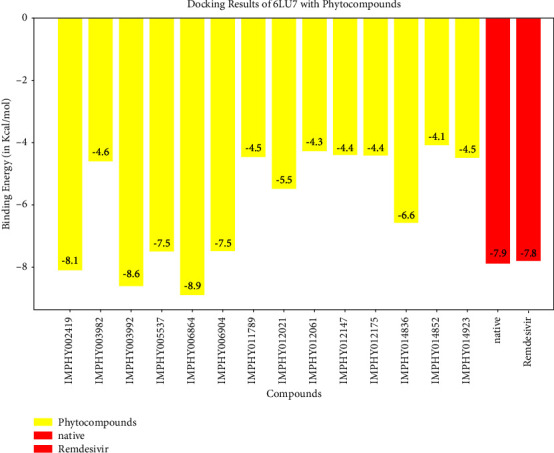
Molecular docking results showing binding energy of phytocompounds with standard compounds against COVID-19 virus.

**Figure 16:**
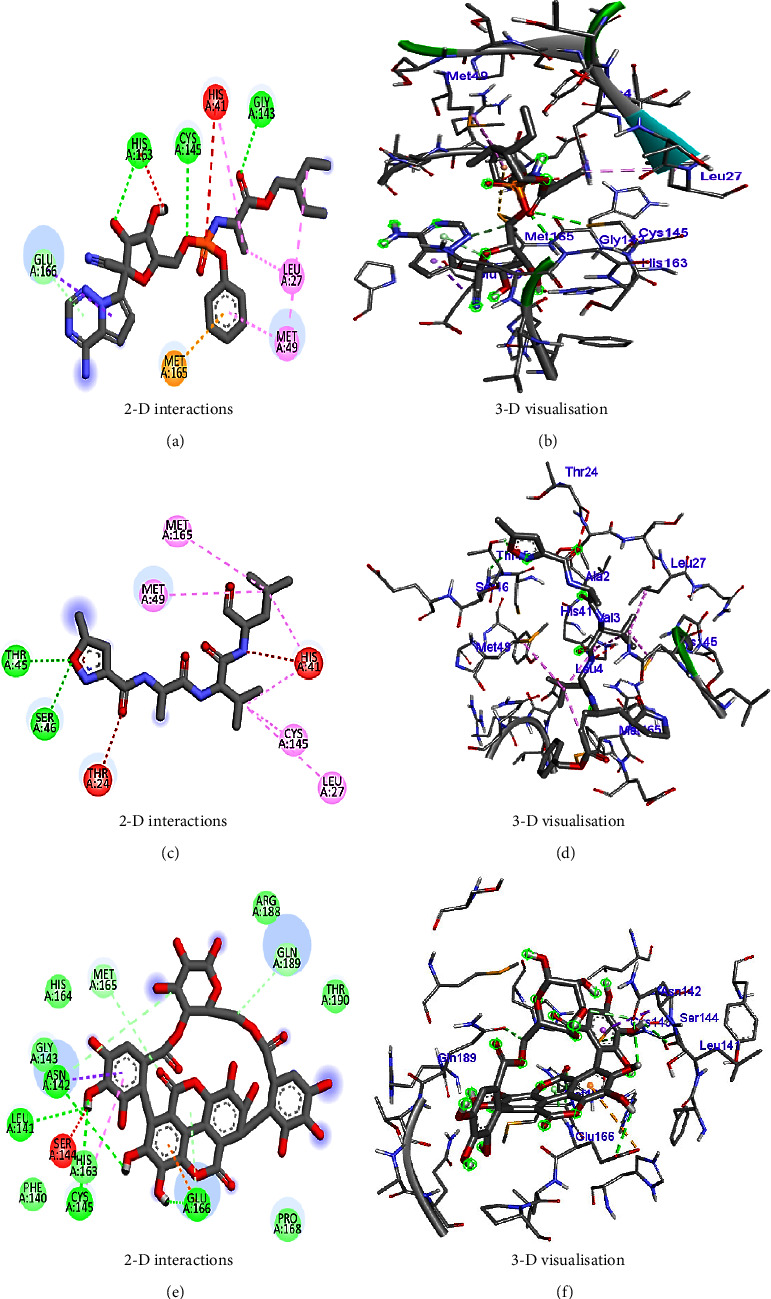
Suggested interaction between 6LU7 and remdesivir (a, b), 6LU7 and native (N3) (c, d), and 6LU7 and IMPHY006864 (e, f).

For COVID-19, the results were compared with the standard compounds remdesivir and native (N3), which exhibited binding energies of −7.8 and −7.9 kcal/mol, respectively (Figure 15 and Table 7). Remarkably, the phytocompound IMPHY006864 (i.e., punicalin, Table 3) expressed a binding energy of −8.9 kcal/mol (Figure 15 and Table 7), surpassing the standard compounds for COVID-19.  Figure 16 shows the suggested interaction. Remdesivir is suggested to form hydrogen bond interactions with three amino acids: HIS A: 163, CYS A: 145, and GLY A: 143. Native (N3) is suggested to form hydrogen bond interactions with two amino acids: THR A: 45 and SER A: 46. Nevertheless, the phytocompound IMPHY006864 suggested forming hydrogen bond interactions with four amino acids: ASN A: 142, LEU A: 141, CYS A: 145, and GLU A: 166. These findings suggest that the phytocompound IMPHY006864 has stronger binding affinities against COVID-19 compared to the standard compounds, accompanied by favourable hydrogen bond interactions with key amino acids in the target protein [37].

## 4. Conclusion

The pomegranate, sweet lime, and lemon peel extracts expressed sufficient antimicrobial activity against the pathogenic Gram −ve *E. coli*. The natural alcohol-free sanitizer formulated from these peel extracts proved its promising efficiency as an antimicrobial agent against the pathogenic *E. coli* and hand microflora. Thus, we recommend its promising contribution to the enhancement of hand hygiene practices and public health.

Moreover, the in silico antiviral testing conducted through molecular docking further bolstered the credibility of the formulation's efficacy. By simulating the interactions between the phytocompounds and target viral proteins, the study provided valuable insights into the potential antiviral activity of the natural alcohol-free sanitizer against COVID-19, influenza, and pneumonia viruses. Through the virtual screening: (1) The phytocompound IMPHY00686 shows promise for further evaluation and potential development as drug-like molecules targeting COVID-19's MPro protein. (2) The phytocompound IMPHY003992 showed that it might be able to effectively block the neuraminidase protein. This shows that IMPHY003992 has a lot of potential as a lead compound for further research and development in the fight against influenza viruses. The emergence of new strains and the continuous threat of influenza outbreaks necessitate the development of effective antiviral therapies. (3) The phytocompound IMPHY002419 has also been discovered through the virtual screening to hold promise as a potent inhibitor of penicillin-binding protein 2B (PBP2B) from *Streptococcus pneumoniae* (strain R6, PDB: 2WAF) and warrants further investigation and development as a potential therapeutic agent against the significant respiratory Gram +ve pathogen *Streptococcus pneumonia*, which is responsible for pneumonia.

Overall, this research contributes to the growing body of knowledge on natural-based alcohol-free hand sanitizers and their antimicrobial properties. The formulation's efficacy against various microbes and in silico antiviral potential presents a compelling case for its use as a natural and sustainable alternative to conventional alcoholic hand sanitizers.

It is essential to conduct a thorough research, testing, and quality control to address the limitations and ensure the safety and effectiveness of herbal hand sanitizers. By addressing the research avenues, future studies can enhance the efficacy, safety, and sustainability of herbal hand sanitizers. In addition, consulting with regulatory authorities and following industry standards are crucial for product development and marketing. Further research studies and clinical trials are warranted to validate its safety and effectiveness for real-world applications.

As the world continues to face challenges posed by infectious diseases, exploring innovative and eco-friendly solutions such as this natural alcohol-free hand sanitizer becomes crucial in promoting public health and well-being. However, there are some challenges and opportunities for application on a large scale. Developing a herbal hand sanitizer made using fruit peels of pomegranate, sweet lime, and lemon has promising real-world applications, but it also presents potential challenges in large-scale production. Fruit peels contain natural compounds that have antimicrobial properties, making them suitable for sanitizing purposes. However, some natural chemicals in fruit peel-derived sanitizers may degrade over time, decreasing their effectiveness. Standardization of ingredients is another challenge; the concentration of active compounds in fruit peels can vary based on factors such as the fruit's origin, ripeness, and processing methods. This can lead to inconsistency in the product's effectiveness. Cost would seasonally vary and would depend on the source and availability of the fruit peels. The collecting and sorting of the fruit peels are a challenge for large-scale production. Moreover, if water or other eco-friendly solvents used for extraction are not recycled and reused, this would add to the cost of the large-scale processes and would cause environmental issues upon the discharge of wasted effluents. The spent waste fruit peels after the extraction and manufacturing of hand sanitizers would cause a waste management problem, so it should be also valorized into other value-added products, for example, activated carbon or biochar with different industrial applications. It can also be valorized into biogas to act as a fuel for the industry. It can also be valorized into organic fertilizer or animal fodder. Upon the valorization of those disposed of spent waste fruit peels, the overall cost of the process would be lowered via the achievement of the circular economy concept. Moreover, ethical considerations, such as ensuring the sustainable sourcing of fruit peels, need to be taken into account to avoid environmental harm. Furthermore, there may be limited scientific research on the specific antimicrobial properties of the readily available fruit peels for hand sanitizers. Thus, more research would be needed to validate and optimize the formulation. Transparent labelling and advertising are also crucial to inform consumers about the ingredients and benefits of the product. A thorough research and testing should be conducted to ensure the effectiveness and safety of the sanitizer. By addressing these considerations, the development and deployment of the fruit peel hand sanitizer can contribute to public health and environmental well-being.

## Figures and Tables

**Table 1:** Phytocompounds present in fruit peels used in the study.

Plant names	Phytocompounds	References
*Punica granatum*	Ellagic acid, punicalagin, gallic acid, fatty acids, quercetin, rutin, flavanols, flavones, flavanones, proanthocyanidins, and anthocyanidins	[20]
*Citrus limetta*	Terpene (d-limonene), geraniol, linalool, phenols, alkaloids, amino acids, anthraquinones, saponin, terpenoid, tannins, flavonoids, flavones, and flavanones	[21]
*Citrus limon*	Beta*-* and *γ*-sitosterol, hesperidin, phenols, alkaloids, saponin, glycosides, terpenoids, tannins, terpenes (d-limonene, citral, and linalool), flavonoids, flavones, flavones, and flavanones	[22]

**Table 2:** Formulation of alcohol-free natural hand sanitizer.

Constituent	Volume (mL)	Volume (g)	Role
Pomegranate peel extract	0.5	0.38	Antimicrobial
Sweet lime peel extract	0.5	0.38	Antimicrobial
Lemon peel extract	0.5	0.38	Antimicrobial
Glycerine	1.0	0.77	Emollient

**Table 3:** The selected 14 phytochemicals for virtual screening and their 2D structures.

Phytochemical identifier	Plant name	Phytochemical name	2D structure
IMPHY006864	*Punica granatum*	Punicalin	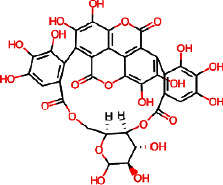

IMPHY006904	*Punica granatum*	Punicalagin	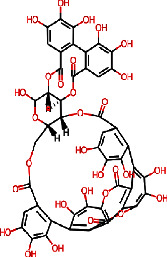

IMPHY002419	*Punica granatum*	Ellagitannin	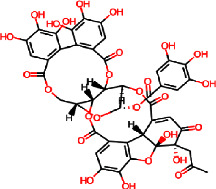

IMPHY012021	*Punica granatum*	Gallic acid	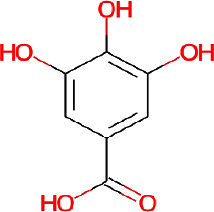

IMPHY005537	*Punica granatum*	Ellagic acid	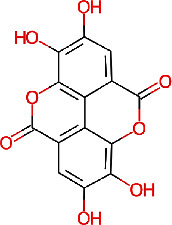

IMPHY012175	*Citrus limetta*	D-limonene	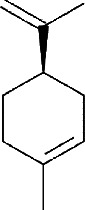

IMPHY014852	*Citrus limetta*	Camphene	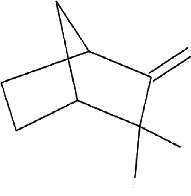

IMPHY012061	*Citrus limetta*	Alpha-pinene	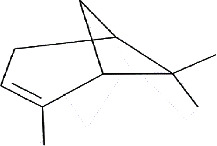

IMPHY012147	*Citrus limetta*	Beta-pinene	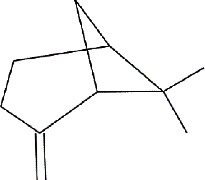

IMPHY014923	*Citrus limetta*	Geraniol	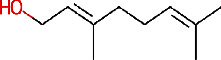

IMPHY003992	*Citrus limon*	Hesperidin	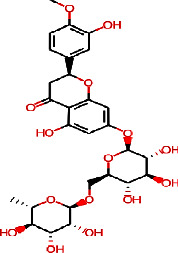

IMPHY014836	*Citrus limon*	Beta-sitosterol	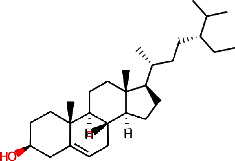

IMPHY003982	*Citrus limon*	Gamma-terpinene	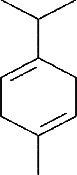

IMPHY011789	*Citrus limon*	Citral	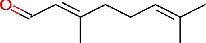

**Table 4:** Yield calculation of different fruit peel extracts.

Sample extract	Solvent	Yield (g/20 g peels)
Pomegranate peels	Ethanol	6.4 ± 0.5
Sweet lime peels	Ethanol	10.8 ± 0.5
Lemon peels	Ethanol	9.5 ± 0.5

**Table 5:** Binding affinity of the phytocompounds against the target receptor of pneumonia virus.

Sample no.	Ligand	Receptor-ligand complex	Binding affinity (kcal/mol)
1	IMPHY002419	2WAF_IMPHY002419	**−10.7**
2	IMPHY003982	2WAF_IMPHY003982	−5.2
3	IMPHY003992	2WAF_IMPHY003992	−9.1
4	IMPHY005537	2WAF_IMPHY005537	−8.5
5	IMPHY006864	2WAF_IMPHY006864	−8.5
6	IMPHY006904	2WAF_IMPHY006904	−8.5
7	IMPHY011789	2WAF_IMPHY011789	−5
8	IMPHY012021	2WAF_IMPHY012021	−5.6
9	IMPHY012061	2WAF_IMPHY012061	−5
10	IMPHY012147	2WAF_IMPHY012147	−5.1
11	IMPHY012175	2WAF_IMPHY012175	−5.2
12	IMPHY014836	2WAF_IMPHY014836	−8.7
13	IMPHY014852	2WAF_IMPHY014852	−5.1
14	IMPHY014923	2WAF_IMPHY014923	−5.2
15	L-Amoxicillin	2WAF_L-amoxicillin	−8.6
16	Penicillin-G	2WAF_penicillin-G	−7
17	Cefprozil	2WAF_cefprozil	−8.1

Bold value represents the highest affinity and relative to the standard compound.

**Table 6:** Binding affinity of the phytocompounds against the target receptor of influenza virus.

Sample no.	Ligand	Receptor-ligand complex	Binding affinity (kcal/mol)
1	IMPHY002419	2HTU_IMPHY002419	−7.2
2	IMPHY003982	2HTU_IMPHY003982	−4.8
3	IMPHY003992	2HTU_IMPHY003992	**−8.4**
4	IMPHY005537	2HTU_IMPHY005537	−7.6
5	IMPHY006864	2HTU_IMPHY006864	−7.8
6	IMPHY006904	2HTU_IMPHY006904	−8.1
7	IMPHY011789	2HTU_IMPHY011789	−4.6
8	IMPHY012021	2HTU_IMPHY012021	−7.8
9	IMPHY012061	2HTU_IMPHY012061	−5.5
10	IMPHY012147	2HTU_IMPHY012147	−5.3
11	IMPHY012175	2HTU_IMPHY012175	−4.9
12	IMPHY014836	2HTU_IMPHY014836	−6.6
13	IMPHY014852	2HTU_IMPHY014852	−5.4
14	IMPHY014923	2HTU_IMPHY014923	−5.1
15	Peramivir	2HTU_peramivir	−5.7

Bold value represents the highest affinity and relative to the standard compound.

**Table 7:** Binding affinity of the phytocompounds against the target receptor of COVID-19 virus.

Sample no.	Ligand	Receptor-ligand complex	Binding affinity (kcal/mol)
1	IMPHY002419	6LU7_IMPHY002419	−8.1
2	IMPHY003982	6LU7_IMPHY003982	−4.6
3	IMPHY003992	6LU7_IMPHY003992	−8.6
4	IMPHY005537	6LU7_IMPHY005537	−7.5
5	IMPHY006864	6LU7_IMPHY006864	**−8.9**
6	IMPHY006904	6LU7_IMPHY006904	−7.5
7	IMPHY011789	6LU7_IMPHY011789	−4.5
8	IMPHY012021	6LU7_IMPHY012021	−5.5
9	IMPHY012061	6LU7_IMPHY012061	−4.3
10	IMPHY012147	6LU7_IMPHY012147	−4.4
11	IMPHY012175	6LU7_IMPHY012175	−4.4
12	IMPHY014836	6LU7_IMPHY014836	−6.6
13	IMPHY014852	6LU7_IMPHY014852	−4.1
14	IMPHY014923	6LU7_IMPHY014923	−4.5
15	Native (N3)	6LU7_native	−7.9
16	Remdesivir	6LU7_remdesivir	−7.8

Bold value represents the highest affinity relative to the standard compound.

## Data Availability

The data used to support the findings of the study are available from the corresponding author upon request.
